# A Study of Inflammatory/Necrosis Biomarkers in the Fracture of the Femur Treated with Proximal Femoral Nail Antirotation

**DOI:** 10.1155/2015/189864

**Published:** 2015-05-14

**Authors:** Mariapaola Marino, Giuseppe Palmieri, Marco Peruzzi, Flavia Scuderi, Emanuela Bartoccioni

**Affiliations:** ^1^Institute of General Pathology, Catholic University, 00168 Rome, Italy; ^2^Department of Orthopaedic Sciences and Traumatology, University Hospital Agostino Gemelli, Catholic University, 00168 Rome, Italy; ^3^Department of Laboratory Medicine, University Hospital Agostino Gemelli, Catholic University, 00168 Rome, Italy

## Abstract

Pertrochanteric fractures are common injuries in adults and source of morbidity and mortality among the elderly. Different surgical techniques were recommended for their treatment but undoubtedly they add an additional inflammatory trauma along the fracture itself. Many attempts to quantify the degree of approach-related trauma are carried out through measurements of systemic inflammatory parameters. In this study we prospectively analyzed laboratory data of 20 patients over eighty with pertrochanteric fracture of the femur treated with proximal femoral nail antirotation (PFNA). This is an excellent device for osteosynthesis because it can be easily and quickly inserted by a mini-incision providing stable fixation and early full mobilization. Serum tumor necrosis factor-alpha (TNF-*α*), interleukin-6 (IL-6), C-reactive protein (CRP), and plasma creatin kinase (CK) were evaluated 1 hour preoperatively and 24 hours postoperatively. Our results show that PFNA did not induce significant increments in serum levels of inflammatory cytokines TNF-*α* and IL-6; CRP was elevated preoperatively in correlation with waiting time for surgery; CRP and CK showed a significant increment in the first postoperatory day; CK increment was correlated with surgical time length. We conclude that, for the markers we analyzed, PFNA shows a low biomechanical-inflammatory profile that represents an advantage over other techniques.

## 1. Introduction 

The absolute number of pertrochanteric fractures may increase in the years to come, along with the increase of population's aging and osteoporosis, with absolute indication for surgery. A surgical intervention as the osteosynthesis of pertrochanteric fracture is a posttraumatic immune stimulus which contributes to the systemic inflammatory response syndrome representing the “second hit” for these patients after the first trauma [1]. Geriatric patients are particularly susceptible to profound inflammatory responses to trauma that, especially if exaggerated, drive mortality and morbidities including infectious complications. Standard laboratory blood analyses are used as first-line tests to determine the preoperative diagnosis of infective/inflammatory status: the erythrocyte sedimentation rate, C-reactive protein (CRP) serum level, and white blood-cell count play a substantial role as markers of inflammation. The extent of fracture and soft-tissue damage can be estimated by analysis of creatine kinase (CK) that is highly predictive with regard to long-term outcome after trauma [2]. Nevertheless these serum markers are not consistently reliable, as they are highly sensitive but less specific because they are affected by the age, the gender, and the medical comorbidities of the patient [3].

The body's response to trauma is a highly complex and heterogeneous sequence of events, and specific cytokine patterns, truly predictive of outcomes, are yet to be established.

Inflammatory cells that contribute to the clearance and the repair of necrotic tissue dominate the local response to injury [4]. These cells release soluble molecules, mainly cytokines that also act on sites distant from the origin of their production while a systemic acute-phase response goes along the local inflammation. This is followed by a compensatory anti-inflammatory response to attenuate the proinflammatory state [5], and the balance between the pro- and anti-inflammatory responses determines the net outcome of the reaction. In major injury, disequilibrium between pro- and anti-inflammatory cytokines may start a generalized response that in turn may progress to a multiple organ dysfunction [6].

Literature evidence suggests that IL-6 levels provide an estimate of not only local tissue trauma but also the subsequent systemic response to trauma [7]. Several studies have focused on the effect of inflammatory cytokines TNF-*α*, IL-6, and IL-1-*β* produced predominantly by activated lymphocytes/mononuclear cells located in inflammatory loci including fracture sites or in bone marrow close to bone cells wherein bone remodeling proceeds. These cytokines are known to be powerful stimulators of bone resorption and to affect bone formation, through complex actions on DNA and collagen synthesis in osteoblastic cells [8]. In particular, while TNF-*α* seems to have contrasting effects on bone formation through osteoblastic differentiation, IL-6 has been shown to be associated with cartilage destruction in human rheumatoid arthritis, to regulate differentiation and apoptosis in preosteoblasts and to affect the mineralization of fracture callus in mice [9].

In case of a major, but standardized, musculoskeletal injury like trochanteric fractures, the total hip replacement induced significant increments in serum levels of the proinflammatory cytokines in the postoperative course, at 6 hours and at 24 hours after surgery [10]. Minimally invasive methods for indirect reduction and fixation try to minimize the impact of the second “inflammatory” hit [11, 12]. Unfortunately, the expected benefit (decreased additional tissue damage) of these minimally invasive techniques [13] has not been objectively measurable.

Recent developments in orthopaedic surgery indicate that pertrochanteric fractures can be successfully treated using advanced implants. Dynamic hip screws (DHSs) and proximal femoral nails (PFNs) are commonly used and both produce good results [14, 15]. Encouraging reports tried to quantify the surgical trauma for pertrochanteric fractures treated by DHSs, based on cytokines [16]: a significant reduction of IL-6 was found in patients undergoing fixation with DHS when comparing a minimally invasive to a more conventional surgical technique. Accordingly, the aim of this study was to determine the second inflammatory hit in patients with fractures of the hip treated with the PFN antirotation (PFNA). This osteosynthesis method combines effectiveness in outcome, easiness of installation, and speed of intervention. We observed minimal inflammatory responses that may be related to a less surgical trauma.

## 2. Materials and Methods

### 2.1. Patients and Surgical Technique

The protocol was approved by the Ethics Committee at our institution. Written informed consent was obtained from each patient enrolled in the study, according to the Declaration of Helsinki.

The clinical data from 20 patients planned for PFNA at the Gemelli Hospital of Catholic University, Department of Orthopaedic Sciences and Traumatology, were collected and analyzed prospectively from November 2011 to September 2012. Inclusion criteria were pertrochanteric fracture of the femur (3.1 type-A according to the AO classification of fractures) (https://www.aofoundation.org, last visited 28/01/2015), investigated by conventional hip RX in the anteroposterior projection and age >65 years. Patients on steroid therapy were excluded from the study because of the anti-inflammatory effects of these drugs.

For each patient, the following was recorded: the age at surgery, the gender, the comorbidity, the preoperative waiting hours, and the operative time (Table 1).

All patients were operated for fracture reduction and fixation with intramedullary nail PFNA, (Synthes, Switzerland) with percutaneous technique. This method involves closed reduction of the fracture by pulling the fractured limb on a special operating table and fluoroscopic control. The nail is inserted into the medullary canal of the proximal femur through a mini-incision of about 10 cm length at the level of greater trochanter. The fixing system involves the insertion of a blade along the femoral neck and up to the head and a screw passing through the two cortical of the femur and the distal portion of the nail. This was performed with a percutaneous technique, which is using two small incisions sufficient to the passage of the screws.

### 2.2. Laboratory Investigations

Samples of venous blood were obtained at 1 hour preoperatively and at 24 hours postoperatively. After centrifugation at 3000 rpm for 5 minutes sera were collected, divided in 3 aliquots for each sample, and stored at −80°C until dosages were performed. They were analyzed anonymously. To check the inflammatory stimulus induced by the intervention blood levels of TNF-*α*, IL-6, and CRP were assayed. The levels of interleukins TNF-*α* and IL-6 were measured by the use of commercial ELISA kits (TNF-*α*, RD Systems, Minneapolis, MN; IL-6, eBioscience, Bender MedSystems, Vienna, Austria). Healthy donors' control values were assumed according to guidelines of the companies, with an average of 15.6 pg/mL for TNF-*α* and 5.8 pg/mL for IL-6. CRP was measured by an immunometric test (Dimension Vista, Siemens Medical Solutions Diagnostics GmbH, Eschborn, Germany) normal value ≤3 mg/L.

Plasma levels of creatine kinase (CK) were measured by absorption photometry (Cobas 8000, Roche Diagnostics, Switzerland), at admittance of the patients in the emergency room and 24 hours after surgery, as indicator of muscle necrosis; normal value ≤190 UI/L.

### 2.3. Statistical Analysis

Data were analyzed using student's *t*-test; *p* values < 0.05 were considered to be significant.

## 3. Results

The patients' clinical features are shown in Table 1. There were 15 females and 5 males, with an average age of 85 (SD 4.5) and 81 (SD 10.8) years, respectively.

The values of serum TNF-*α*, IL-6, CRP, and CK are shown in Table 2. The mean levels (±1 SD) of TNF-*α* were 2.27 ± 4.09 pg/mL preoperatively and 3.84 ± 5.74 pg/mL after 24 hours postoperatively; these differences were not statistically significant.

The preoperative mean level of IL-6 was 16.14 ± 14.96 pg/mL, nearly threefold the value of normality though with a very wide range of variation, while the mean level after 24 hours postoperatively was 16.64 ± 9.04 pg/mL: this difference was not statistically significant.

Before surgery, the CRP level was high in all patients (>3 mg/L) with a very wide range of variation, 63.08 ± 33.72 mg/L. The mean level after 24 hours postoperatively, 104.46 ± 38.69 mg/L, demonstrated a statistically significant increase (*p* = 0.000047).

**Figure 1:**
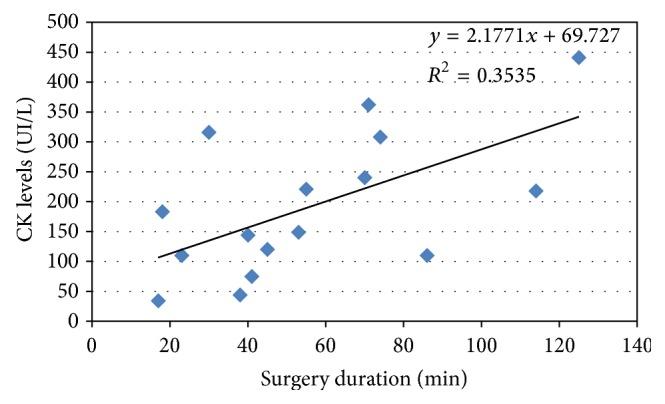
Correlation between CK levels and duration of the surgery.

The preoperative plasma CK was within the normal range (30–190 UI/L) for all patients, with a mean value of 84.35 ± 44.81 UI/L. There was a statistically significant increase during the first 24 postoperative hours (with a mean value of 218.06 ± 155.93 U/L, pre versus post *p* = 0.001), in spite of a wide range of variation for CK. After 24 h after surgery, CK plasma levels of only eight patients greatly exceeded the normal range. The postoperative levels were positively significantly related to the surgical time length (*R* ^2^ = 0.3535).  Figure 1 shows these results for the 16 patients of whom these data were available.

## 4. Discussion

Fractures of the proximal femur and hip are relatively common injuries in adults and common source of morbidity and mortality among the elderly. Incidence of fractures is increasing, which is not unexpected because the general life expectancy of the population has increased significantly during the past few decades. Many methods for the treatment of pertrochanteric fractures have been recommended. PFNA is an excellent device for osteosynthesis, as it can be easily inserted. Moreover, it provides for stable fixation, which allows early full mobilization of the patient [17]. Much research is now being conducted in order to understand the role of cytokines in the development of the inflammatory response following the surgical trauma.

On this background, the aim of this study was to evaluate the second inflammatory hit, correlated to soft-tissue invasiveness, in 20 patients with fractures of the hip treated in our institution with PFNA.

To this purpose, we analyzed the changes in plasma levels of the inflammatory cytokines TNF-*α* and IL-6, along with conventional systemic markers CRP and CK.

The markers analyzed varied widely between studies. IL-6, a major proinflammatory cytokine, is mainly produced by monocytes and activated macrophages, even though other cells (such as fibroblasts and myoblasts) may also synthesize it [18, 19]. Surgery may instantly mediate IL-6 release from these cells, or IL-6 release may be induced by other locally released cytokines [20, 21]. Experimental data demonstrated that TNF-*α* induces IL-6 secretion in human myoblasts, the* in vitro *counterparts of the regenerating muscle cells that, following a muscle injury/trauma, develop from satellite cells, proliferate, fuse, and differentiate into new mature myofibers [22]. Furthermore, IL-6 is principally responsible for activating the hepatic synthesis of CRP, which has been considered the inflammatory biomarker of choice in orthopaedic surgery [23, 24].

In our study, waiting time before surgery ranged from 99 h 41′ to 14 h 33′ (average 45 h 30′), which is sufficient for cytokines to become detectable in serum [20]. The total duration of surgery ranged from 17 to 71 minutes (average 36′). We analyzed for TNF-*α*, IL-6, and CRP the patient sera collected 1 h before and 24 h after surgery. In the case of elevated cytokine concentrations at admission time, some authors reported that injured patients with high concentrations of IL-6 and TNF-*α* showed an increased risk of death [6]. In our patients TNF-*α* was not detectable before surgery, without significant changes in the postoperative 24 hours. In keeping with previous reports, TNF-*α* is probably unuseful for the purpose of quantifying surgical tissue damage [16, 25]; in any case, the minimal surgical trauma induced by PFNA was not sufficient to display an appreciable increase in serum levels of this cytokine.

Before surgery, IL-6 was detectable in 16/20 patients with an average value that was nearly threefold the value of normality, without correlation with waiting time; the maximum value was 45.9 pg/mL in a patient who had waited only 14 hours. However also for IL-6 we did not find any statistically significant difference between the preoperative and the postoperative average value (16.14 ± 14.96 versus 16.64 ± 9.04 pg/mL), even with a very wide range of variation. Since injured muscle tissue also produces IL-6, these data underline that the first hit (trauma) is able to cause an increase of IL-6, while osteosynthesis PFNA method does not lead to a further soft tissue damage [18]. Other authors described IL-6 production 1 hour after surgery, comparing conventional (conv) and minimally invasive (MI) DHS techniques: the preoperative levels of IL-6 recorded showed no significant differences between the two groups, whereas postoperative values were on average higher in the DHS conv group (78.41 ± 67.04 pg/mL) compared with the DHS MI group (39.02 ± 37.36 pg/mL) [16]. We selected the 24 hours' time point instead of 1 hour after surgery because in our experience it represents the best time range to observe IL-6 production peak following inflammatory stimuli [22, 26].

CRP is an acute phase protein that can be used as a marker for changes in the orthopedic postoperative inflammatory response and its levels may depend on the region of trauma. CRP levels increase rapidly after surgery, peaking on day 2, but already after 24 hours the increase starts to decline to plateau [27]. We found that the CRP values were increased, as measured 1 hour preoperatively and that there was a further increase of about 60% 24 hours after surgery (from 63.08 ± 33.72 to 104.46 ± 38.69 mg/L, *p* = 0.000047). Although not significant, there was also a positive association trend between the values of CRP and preoperative waiting hours (data not shown). CRP levels may also be normal preoperatively but consistently increase postoperatively with various types of fracture fixation. Our results are in agreement with literature: Hong and coworkers found similar results for CRP analyzing serum soft-tissue marker differences after PFN fixation of stable pertrochanteric fractures in the elderly [28].

In addition, many reports have confirmed CK as a possible indicator of soft tissue damage during surgery. CK changes in plasma have been reported as a result of a skeletal muscle injury and correlated with surgical incision length, approach, and operative time. A trend of CK increase was observed in elderly patients undergoing surgery for hip fracture with the peak values on day 1 postoperatively [29, 30]. In our patients the preoperative plasma CK level was in the normal range with an average value of 84.35 ± 44.81 UI/L but it increased significantly during the first 24 postoperative hours, reaching a mean value of 218.06 ± 155.93 UI/L. Interestingly, only for 8/20 patients CK greatly exceeded the normal range with a very significant positive correlation to the surgery time, which may mean major muscle damage and inflammation. The mean CK value that we found 24 h postoperatively is in accordance with literature on minimally invasive hip surgery [31].

To analyse and compare serum soft-tissue marker differences after the minimal invasive DHS and PFN fixation, Hong and coworkers found that both methods showed similar patterns of change for CK and CRP pre- and postoperatively (up to 72 h) without significative differences, suggesting that both approaches produce similar levels of soft-tissue damage [28]. Our results on PFN are similar to those reported by Hong, even if the values are expressed in different units.

In conclusion, in the treatment of pertrochanteric fracture, secretion of inflammatory markers is lower when minimally invasive techniques are used compared with traditional surgery. Good results have been reported for intramedullary devices. We confirm that, for the markers analyzed here, PFNA has a low biomechanical-inflammatory profile that represents an advantage over other techniques. Above all, it depends on a short surgical time. Average time for the synthesis of a trochanteric fracture by a skilled operator is 25 minutes (by incision to suture). The reduced surgical time together with the minimal surgical incision, not longer than 10 cm, represents the useful features of this method. Further, closed reduction of the fracture performed under fluoroscopic control avoids a second incision at the level of the fracture. This allows a minimum of trauma to soft tissue, resulting in a lower inflammatory stimulus compared to methods that involve exposure of the fracture site.

## Figures and Tables

**Table 1:** Clinical features of the studied patients.

Patients, number	20

Gender	15 females, 5 males

Mean age, years, range	84 (68–94)

Time from hospitalization to surgery (hours, range)	45 h 30′ (99 h 41′–14 h 33′)

Total duration of surgery (minutes, ranges)	36′ (17′–71′)

**Table 2:** It shows the data of the cytokines TNF-*α* and IL-6 and of the markers CRP and CK obtained from serum or plasma samples collected 1 hour before and 24 hours after surgery. *p* < 0.05 was considered significant.

	Surgery	Range	Mean value ± Standard deviation	*p* value
TNF-*α* (pg/mL)	pre	0.1–14.3	2.27 ± 4.09	n.s.
	post	0.1–18.3	3.84 ± 5.74	

IL-6 (pg/mL)	pre	0.1–45.9	16.15 ± 14.96	n.s.
	post	0.1–32.7	16.64 ± 9.04	

CRP (mg/L)	pre	14.2–111.0	63.08 ± 33.72	*p* = 0.000047
	post	29.8–162.0	104.46 ± 38.69	

CK (UI/L)	pre	24.0–230.0	84.35 ± 44.81	*p* = 0.001
	post	34.0–632.0	218.06 ± 155.93	
